# The mTOR Deficiency in Monocytic Myeloid-Derived Suppressor Cells Protects Mouse Cardiac Allografts by Inducing Allograft Tolerance

**DOI:** 10.3389/fimmu.2021.661338

**Published:** 2021-04-09

**Authors:** Jiawei Li, Juntao Chen, Mingnan Zhang, Chao Zhang, Renyan Wu, Tianying Yang, Yue Qiu, Jingjing Liu, Tongyu Zhu, Yi Zhang, Ruiming Rong

**Affiliations:** ^1^ Department of Urology, Zhongshan Hospital, Fudan University, Shanghai, China; ^2^ Shanghai Key Laboratory of Organ Transplantation, Shanghai, China; ^3^ Department of Immunology and Microbiology, Shanghai Institute of Immunology, Shanghai Jiao Tong University School of Medicine, Shanghai, China; ^4^ Department of Critical Care Medicine, Zhongshan Hospital, Fudan University, Shanghai, China; ^5^ Biomedical Research Center, Institute for Clinical Sciences, Zhongshan Hospital, Fudan University, Shanghai, China

**Keywords:** MDSC (myeloid-derived suppressor cell), cardiac transplantation, mTOR, autophagy, immune tolerance

## Abstract

**Background:**

Myeloid-derived suppressor cells (MDSCs) can prevent allograft rejection and induce immune tolerance in transplantation models. Previous studies have demonstrated that inhibition of mTOR signaling can enhance the MDSC protective effect in heart transplantation (HTx) by promoting MDSC expansion. In addition, mTOR inhibition is related to autophagy. The present study investigated the protective mechanism of mTOR-deficient monocytic MDSCs (M-MDSCs) in mouse HTx.

**Methods:**

Myeloid-specific mTOR conditional knockout mice were generated to obtain mTOR^−/−^ M-MDSCs. The proliferation and immunosuppressive function of mTOR^−/−^ M-MDSCs were determined by flow cytometry and T cell proliferation assays. The mTOR^−/−^ M-MDSC intracellular autophagy levels were determined using western blotting and electron microscopy. RNAseq analysis was performed for wild-type (WT) and mTOR^−/−^ M-MDSCs. Allogeneic HTx mouse model was established and treated with WT or mTOR^−/−^ M-MDSCs. Enzyme-linked immunosorbent assay, flow cytometry, and immunohistochemistry assays were performed to determine WT and mTOR^−/−^ M-MDSC-induced immune tolerance.

**Results:**

The mTOR deficiency promoted M-MDSC differentiation and enhanced intracellular autophagy levels *in vivo* and *in vitro*. mTOR deficiency also enhanced the immunosuppressive function of M-MDSCs. In addition, infusing with WT and mTOR^−/−^ M-MDSCs prolonged cardiac allograft survival and established immune tolerance in recipient mice by inhibiting T cell activation and inducing regulatory T cells.

**Conclusion:**

mTOR deficiency enhances the immunosuppressive function of M-MDSCs and prolongs mouse cardiac allograft survival.

## Introduction

Myeloid-derived suppressor cells (MDSCs) were first discovered in tumorigenesis, but were later found to be involved in other pathological conditions, such as obesity, sepsis, and organ transplantation (1). MDSCs are heterogeneous progenitor and immature myeloid cells. Based on the different cell surface markers, MDSCs can be classified into granulocytic MDSCs (G-MDSCs and CD11b^+^Ly6G^+^Ly6C^low^) and monocytic MDSCs (M-MDSCs and CD11b^+^Ly6G^-^Ly6C^high^) (1, 2). MDSCs are components of tumor microenvironment (TME) and support tumor progression, invasion, and metastases (3, 4). In TME, MDSCs suppress T cell activation, regulate inflammatory cytokines, and promote immune tolerance to enhance tumor growth (5). The function of T cell inhibition by MDSCs is correlated with the induction and recruitment of regulatory T cells (Tregs) (2, 6, 7).

Some recent studies have found that infusing with *in vitro*-induced MDSCs can prevent rejection in cardiac transplantation and recruitment for chimerism and tolerance (8). Preclinical studies have demonstrated that murine MDSCs, derived from either embryonic stem cells or adult bone marrow (BM), potently inhibit graft-versus-host disease without aborting graft-versus-leukemia response (9). MDSCs treated with drugs or cytokines, or edited using genetic technology *in vitro*, showed directional differentiation potential or enhanced function in inducing tolerance. In mouse skin transplantation models, M-CSF plus TNF-α-induced M-MDSCs have a stronger immunosuppressive function and promote immune tolerance against donor antigens (10), as well as CsA-induced MDSCs (11). In addition, prolonged mouse cardiac allograft survival was demonstrated after treatment with mammalian target of rapamycin (mTOR) inhibitor rapamycin, which induced MDSC expansion in recipient hearts (12). Our previous study demonstrated that blocking the mTOR signaling pathways ameliorated acute kidney injury (AKI) by promoting the induction, recruitment, and immunosuppressive activity of MDSCs (13).

The inhibition of mTOR is related to autophagy (14). Autophagy is initiated when mTOR is downregulated, resulting in the release of the transcription factor Unc-51-like autophagy-activating kinase 1, which, in turn, induces vacuole formation by activating PI3KC3, Beclin1 (Atg6), and the Atg12, -5, and -16 complex (15). The influence of mTOR inhibition on MDSC proliferation/function has been recently elucidated by Wu et al. in alloskin-grafted and tumor-bearing mouse models. They demonstrated that rapamycin treatment or using mTOR knockout (KO) mice decreased the percentages and number of M-MDSCs and reduced their immunosuppressive activity (16). Autophagy inhibition also increased the quantity of apoptotic MDSCs, demonstrating that autophagy extends the survival and increases the viability of MDSCs (17). Whether mTOR deficiency may affect MDSC differentiation in cardiac transplantation remains unknown.

The present study found that mTOR deficiency can promote M-MDSC differentiation and enhance intracellular autophagy levels, which leads to the upregulated immunosuppressive function of M-MDSCs. In addition, infusing with mTOR-deficient M-MDSCs can prolong cardiac allograft survival and establish immune tolerance in recipient mice by inhibiting T cell activation and inducing Tregs. These findings can potentially be used in the future for preventing graft rejection and immune tolerance induction in transplantation.

## Materials and Methods

### Mice

Wild-type (WT) C57BL/6 and BALB/c mice were purchased from the SLAC Laboratory Animal Co., Ltd (Shanghai, China). Lyz-Cre and mTOR^flox/flox^ mice were generously provided by Professor Yong Zhao at the Institute of Zoology, Chinese Academy of Sciences (Beijing, China). Myeloid-specific mTOR conditional knockout (KO) mice (mTOR^−/−^, Lyz-Cre) were generated by mating mTOR^flox/flox^ mice with Lyz-Cre mice. Mouse genotypes were determined using PCR with the following primers: Lyz-common, Lyz-WT, Lyz-mutant, mTOR-common, mTOR-WT, and mTOR-mutant. Primer sequences are listed in **Table 1**. All animal experiments were performed according to the guidelines of the Care and Use of Laboratory Animals of the Laboratory Animal Ethical Commission of Fudan University and were approved by the Animal Ethical Committee of Zhongshan Hospital, Fudan University, Shanghai, China.

**Table 1:** Genotyping primer list.

Primers’ name	Primers’ sequence
Lyz-common	CTTGGGCTGCCAGAATTTCTC
Lyz-mutant	CCCAGAAATGCCAGATTACG
Lyz-wt	TTACAGTCGGCCAGGCTGAC
mTOR mutant	TCGTGGTATCGTTATGCGCC
mTOR common	CAGCCCCTTGGTTCTCTGTC
mTOR wt	ACAAGGCTCATGCCCATTTC

For genotyping mTOR-flox mouse, we used mTOR-common as forward primer, and both mTOR-wt and mTOR-mutant as reversed primers. If mTOR was not knocked out, the primer pair mTOR-common and mTOR-wt will work and amplifying DNA band at desired size, while the primer pair mTOR-common and mTOR-mutant did not work. If mTOR was knocked out, the primer pair mTOR-common and mTOR-mutant will work and amplifying DNA band at desired size, while the primer pair mTOR-common and mTOR-wt did not work. The genotyping Lyz-Cre mouse was constructed by using the same way.

### Cervical Cardiac Transplantation Model

Allogeneic heterotopic heart transplantation (HTx) was performed as previously described (18). Briefly, cardiac grafts from BALB/c donor mice were perfused with cold heparinized normal saline (4°C) and stored at 4°C for 10–15 min. The common carotid artery and the external jugular vein of C57BL/6 recipient mice were ligated. Then, the two vessels were everted onto two cuffs. Donor ascending aorta and pulmonary artery were anastomosed end-to-end to the recipient’s common carotid artery and external jugular vein. The graft function was evaluated daily by observation of donor heartbeat palpitation. Graft rejection was defined as complete cessation of the heartbeat.

Recipient mice were divided into four groups (1): syngeneic control group, mice transplanted with isografts (C57BL/6 to C57BL/6); (2) allogeneic HTx group, mice transplanted with allografts (BALB/c to C57BL/6); (3) WT M-MDSC group, allogeneic HTx mice treated with WT MDSCs; and (4) mTOR^−/−^ M-MDSC group, allogeneic HTx mice treated with mTOR^−/−^ MDSCs. Recipient mice from MDSC treatment groups were intravenously treated with 5 × 10^5^ of MDSCs through the tail vein 1 day prior to HTx.

### Primary MDSC Culture

The generation of BM-derived MDSCs was performed as previously described (13). Briefly, BM was flushed from femurs and tibias with phosphate-buffered saline (PBS). Red blood cells were lysed with lysis buffer (BD Biosciences, Franklin Lakes, NJ, USA). Then, cells were incubated in dishes for 2 h and non-adherent cells were collected and cultured with 50 ng/ml of GM-CSF and 50 ng/ml of IL-6 in RPMI 1640 medium with 10% fetal bovine serum, 1% MEM non-essential amino acid solution, 1% sodium pyruvate, 1% penicillin–streptomycin–glutamine (all from Gibco, NY, USA), and 2 μl of 2-mercaptoethanol (Sigma-Aldrich, St. Louis, MO, USA) for 7 days at 37°C and 5% CO_2_.

### Flow Cytometry and Cell Sorting

To analyze MDSCs, single-cell suspensions were prepared from peripheral blood, spleens, and hearts. Collected cells were labeled with CD11b (PerCp-cy5.5), Ly6C (FITC), and Ly6G (all from eBioscience, San Diego, CA, USA) and analyzed on BD FACSAria™ II (BD Biosciences). Cell sorting was performed on BD FACSAria™ III Cell Sorter (BD Biosciences).

### CFSE T Cell Proliferation Assays

CD4^+^ T cells were magnetically purified from spleens of WT C57BL/6 mice in accordance with the manufacturer recommendations (Miltenyi Biotec, Auburn, CA, USA). Then, CD4^+^ T cells were stimulated with concanavalin A (ConA; 50 μg/ml; Sigma-Aldrich) and labeled with CFSE (2.5 μM; Invitrogen, USA). A total of 1×10^5^ CFSE-labeled CD4^+^ T cells were co-cultured with G-MDSCs or M-MDSCs at a ratio of 1:1, 2:1, or 4:1 in 96-well round bottom plates. After 3 days, the CFSE signal of gated CD4^+^ T cells was analyzed by flow cytometry on BD FACSAria™ II (BD Biosciences).

### Histological and Immunohistochemical Analysis

Heart specimens were fixed in 10% formalin, embedded in paraffin, and cut into 5-μm sections. Then, sections were deparaffinized and stained with hematoxylin and eosin (H&E) at room temperature. For immunohistochemical staining, sections from heart specimens were incubated with the primary antibody against mTOR and then labeled with fluorescence-conjugated secondary antibodies. Sections were then washed, dried, sealed, and examined under a microscope (Leica, Wetzlar, Germany).

### Quantitative Real-Time PCR

Total RNA was extracted from cells and was subsequently reverse-transcribed using Superscript II reverse transcriptase and random primer oligonucleotides (Vazyme Biotech Co., Ltd., Nanjing, China). Quantitative real-time PCR was performed using HieffTM qPCR SYBR Green Master Mix (Yeasen, Shanghai, China) on an ABI Prism 7900HT (Applied Biosystems, Foster City, CA, USA). The thermocycler conditions included a 2-min incubation at 50°C, followed by an incubation at 95°C for 10 min. This was followed by a two-step PCR program as follows: 15 s at 95°C and 60 s at 60°C for 40 cycles. GAPDH was used as an internal control to normalize the differences in the amount of total RNA in each sample. The primers for the genes of interest were as follows (5′–3′): mTOR, CAG TTC GCC AGT GGA CTG AAG and GCT GGT CAT AGA AGC GAG TAG AC; GAPDH, AGG TCG GTG TGA ACG GAT TTG and TGT AGA CCA TGT AGT TGA GGT CA.

### Western Blotting

For western blotting analyses, MDSCs sorted using flow cytometry were washed with cold PBS and lysed in RIPA buffer (Sigma-Aldrich) on ice for 30 min. Lysates were centrifuged at 15,000×*g* for 10 min at 4°C. A total of 40 μg of cell protein were separated by sodium dodecyl sulfate-10% polyacrylamide gel electrophoresis and transferred to a nitrocellulose membrane (Millipore, Tullagreen, Ireland). After blocking with TBS and 2% milk for 1 h at room temperature, membranes were incubated overnight at 4°C with the following diluted primary antibodies: Beclin1, Atg5, Atg7, LC3I/II, and β-actin (all from CST, Danvers, MA, USA; dilution: 1:1,000). After extensive washing, membranes were incubated for 1 h at room temperature with the corresponding HRP-coupled secondary antibodies. Immunoreactive proteins were visualized using an ECL reagent.

### Enzyme-Linked Immunosorbent Assay (ELISA)

Serum samples were isolated by centrifuging blood at 1,000×*g* for 10 min at 4°C. The samples were diluted nine times with PBS (pH 7.4). A total of 40 µl of diluted serum sample were placed in each well of the 96-well plate coated with the following antibodies: IL-1β, IFN-γ, TNF-α, and IL-1β. The plate was then incubated with 100 µl of HRP-labeled antibodies for 60 min at 37°C. The reactions were then stopped and the OD values at 450 nm were recorded.

### Electron Microscopy

For electron microscopy analyses, MDSCs were fixed in 2% PFA and 2.5% glutaraldehyde in 0.1 M sodium cacodylate buffer (Sigma-Aldrich; pH 7.4) for 2 h on ice. After three washes with 0.1 M PBS at pH 7.4, the cells were fixed with 1% osmium tetroxide in 0.1 M PBS at pH 7.4 for 2 h. The cells were then dehydrated and embedded in Epon resin following a standard procedure as previously described. Subsequently, 70-nm sections were obtained using an UC7 ultramicrotome (Leica) and stained with uranyl acetate and lead citrate. Cell sections were analyzed using an electron microscope.

### RNA Sequencing and Bioinformatics Analysis

WT and mTOR^−/−^ M-MDSCs were obtained from WT and Lyz-mTOR mice (n = 3). Total RNA samples were isolated using TRIzol (Sigma-Aldrich). RNA quality was determined using the ratios of A260/A280 with a Nanodrop 2000 spectrophotometer (Thermo Fisher Scientific, Waltham, MA, USA). RNA samples were further tested using an Agilent 2100 Bioanalyzer (Agilent Technologies, USA). Samples with RIN>7 were selected for sequencing library construction using the (Illumina, USA). The final library was sequenced using an Illumina Novaseq 6000 sequencer. On average, about 20 million 150-bp paired end reads were generated per sample. The raw reads were aligned to the mouse reference genome (version mm10) using Bowtie2 RNASeq alignment software. Then, HTSeq was used to generate read counts for every gene. The read counts were normalized using DESeq2. Subsequently, the normalized read counts were centered and scaled for each gene, generating z-scores (**Table 2**). A Benjamini–Hochberg corrected p-value of <0.05 and fold-change of at least 2 were considered significant. Gene ontology (GO) and Kyoto Encyclopedia of Genes and Genomes (KEGG) analyses were performed using OmicsBean software.

**Table 2:** Raw data size.

Libraries’ name	Raw bases (G)	Raw reads (M)
L-M1	5.74	19.13
L-M2	6.02	20.07
L-M3	6.40	21.33
WT1	6.43	21.43
WT2	6.76	22.53
WT3	6.30	21.00

1 M raw reads = 0.3 G raw bases.

### Statistical Analysis

Data were analyzed using GraphPad Software (GraphPad Holdings, San Diego, CA, USA). Student’s *t-*test was used to analyze the differences between two groups. One-way ANOVA was used to analyze whether an overall statistically significant change existed using a two-tailed paired or unpaired Student’s *t* test. All analyses were performed at least three times independently and results were expressed as the mean ± SD. A p-value of <0.05 was considered statistically significant.

## Results

### The mTOR Deficiency Enhances Endogenous MDSC Differentiation and the Immunosuppressive Function

**Figure 1:**
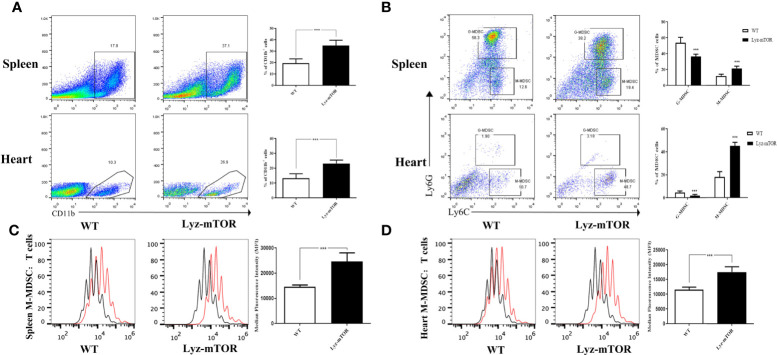
Enhanced M-MDSC differentiation and immunosuppressive function in Lyz-mTOR mice. **(A)** Percentages of CD11b^+^ cells in spleens and hearts from WT and Lyz-mTOR mice. **(B)** Percentages of G-MDSCs and M-MDSCs in spleens and hearts from WT and Lyz-mTOR mice. **(C, D)** CFSE-labeled T cell proliferation pattern was examined using flow cytometry analysis of CFSE dilution in gated CD4^+^ T cells. CFSE MFI represents T cell proliferation, which reflects the immunosuppressive function of G-MDSCs and M-MDSCs in spleens and hearts from WT and Lyz-mTOR mice (n = 5 mice per group, ^***^P < 0.001).

After verifying the depletion of mTOR in MDSCs (**Supplementary Figure 1**), we investigated whether mTOR deficiency had an effect on MDSC differentiation and function, spleen- and heart-derived MDSC populations were detected in WT and Lyz-mTOR mice. Flow cytometry analysis showed that heart and spleen CD11b^+^ cell populations from Lyz-mTOR mice were significantly increased (**Figure 1A**). The mTOR deficiency also increased the percentage of M-MDSCs, but decreased the G-MDSC ratio (**Figure 1B**). Moreover, M-MDSCs from Lyz-mTOR mice showed a stronger immunosuppressive function in inhibiting CD4^+^ T cell proliferation than M-MDSCs from WT mice (**Figures 1C, D**). These results suggested that mTOR deficiency enhanced M-MDSC proliferation and the immunosuppressive function *in vivo*.

### The mTOR Deficiency Promotes M-MDSC Proliferation and Immunosuppressive Function *In Vitro*

**Figure 2:**
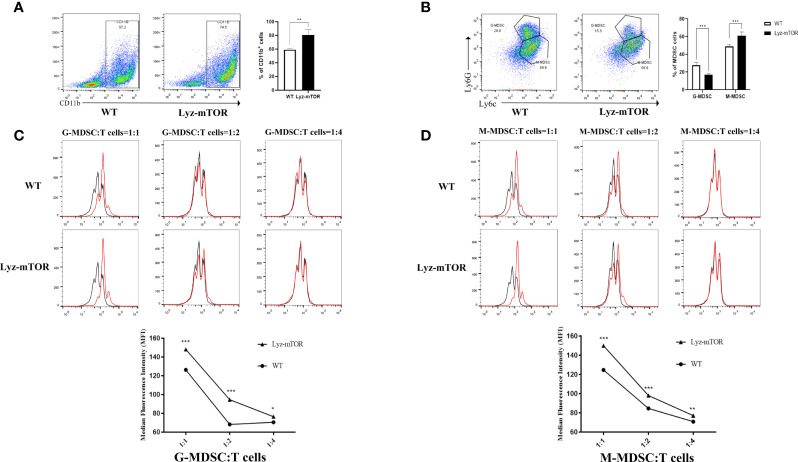
Increased induced M-MDSC ratios and immunosuppressive functions in Lyz-mTOR mice. **(A)** Percentages of CD11b^+^ cells from WT and Lyz-mTOR mouse BM. **(B)** Percentages of G-MDSCs and M-MDSCs from WT and Lyz-mTOR mice. **(C, D)** Induced G-MDSCs and M-MDSCs were co-cultured with T cells stimulated by ConA (G/M-MDSCs: T cells = 1:1, 1:2, and 1:4). CFSE-labeled T cell proliferation pattern was examined using flow cytometry analysis of CFSE dilution in gated CD4^+^ T cells. G/M-MDSCs inhibitory curves were generated (n = 5, ^*^P < 0.05, ^**^P < 0.01, and ^***^P < 0.001).

To confirm whether mTOR deficiency has an influence on BM-induced MDSC differentiation and immunosuppressive function *in vitro*, BM samples from WT and Lyz-mTOR mice were harvested for MDSC induction. Flow cytometry analysis indicated that mTOR deficiency dramatically increased the percentage of CD11b^+^ cells (**Figure 2A**) and M-MDSCs, but decreased the G-MDSC ratio (**Figure 2B**) in BM from Lyz-mTOR mice. To determine the role of mTOR in immunosuppressive function of MDSCs, T cell proliferation was elicited using ConA with or without BM-derived MDSC*** from WT and Lyz-mTOR mice. Both mTOR^-/-^ G-MDSCs and M-MDSCs markedly inhibited the proliferative response of CD4^+^ T cells in a dose-dependent manner (**Figures 2C, D**). According to these results, mTOR deficiency shifted the differentiation from G-MDSCs towards M-MDSCs and significantly enhanced the immunosuppressive function of BM-derived G-MDSCs and M-MDSCs *in vitro*.

### The mTOR Deficiency Enhances Intracellular M-MDSC Autophagy Levels

**Figure 3:**
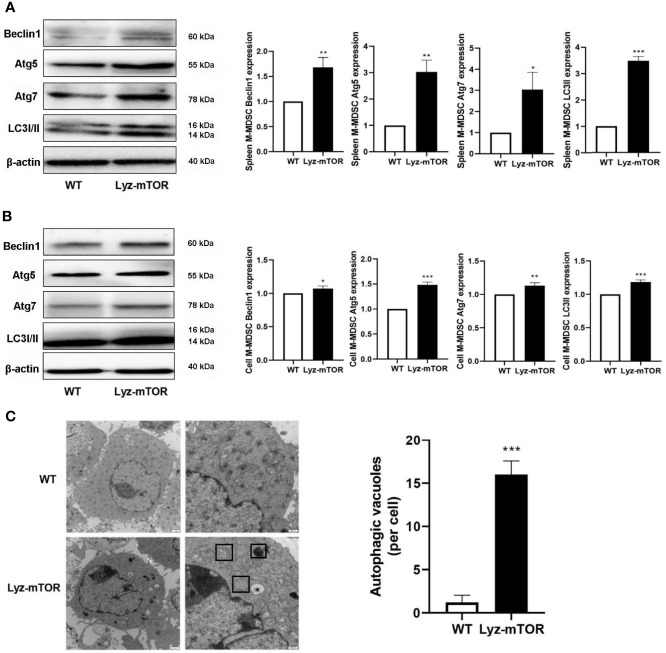
Enhanced autophagy levels in M-MDSCs. **(A, B)** Spleen/induced M-MDSC intracellular autophagy-related proteins, such as Beclin1, Atg5, Atg7, and LC3I/II, were detected by western blot and normalized to β-actin. **(C)** Autophagic vacuoles were evaluated by electron microscopy (n = 5, ^*^P < 0.05, ^**^P < 0.01, and ^***^P < 0.001).

Next, autophagy levels in endogenous and BM-induced M-MDSCs were detected. M-MDSCs from the spleens of WT and Lyz-mTOR mice were harvested and intracellular autophagy-related proteins were examined. Western blot analysis showed that the expression of Beclin1, Atg5, Atg7, and LC3I/II was significantly increased in mTOR^−/−^ M-MDSCs (**Figure 3A**). Accordingly, similar results for the expression of intracellular autophagy-related proteins in BM-induced M-MDSCs with mTOR deficiency (**Figure 3B**) were obtained. Furthermore, electron microscopy was used to investigate the intracellular autophagy levels. More autophagic vacuoles were present in mTOR^-/-^ M-MDSCs than in WT M-MDSCs (**Figure 3C**). This finding demonstrated that mTOR deficiency enhanced intracellular M-MDSC autophagy levels.

### The mTOR^−/−^ M-MDSCs Have the Potential to Reduce Allograft Rejection

**Figure 4:**
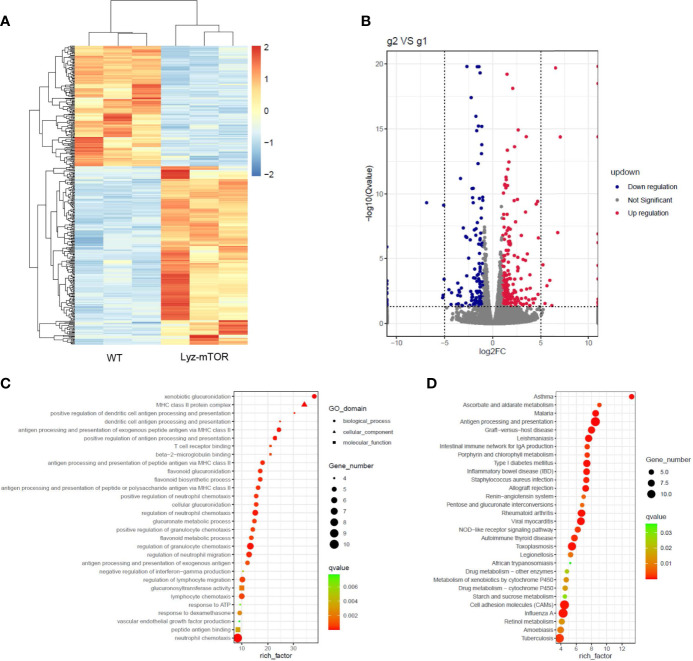
RNAseq analysis of WT and mTOR^−/−^ M-MDSCs: DEGS, GO enrichment, and KEGG pathway. **(A)** DEG heat map. **(B)** DEG volcano plot. **(C)** DEG GO enrichment analysis, including biological process, cellular component, and molecular function. **(D)** Main KEGG pathways (n = 3 per group).

RNA sequencing (RNAseq) was employed to investigate differentially expressed genes (DEGs) between WT and mTOR^−/−^ M-MDSCs. Down-regulated genes were colored blue and up-regulated genes were colored red (**Figure 4A**). The volcano plot showed DEGs up- or down-regulated by mTOR deficiency (**Figure 4B**). GO enrichment analysis identified the main DEG functions, including T cell receptor binding (**Figure 4C**). Furthermore, DEG KEGG pathways showed that allograft rejection is involved in the KEGG pathways (**Figure 4D**), implying that mTOR^-/-^ M-MDSCs might have a role in suppressing T cell activation and reducing rejection in organ transplantation.

### The mTOR^−/−^ M-MDSC Significantly Prolongs Cardiac Allograft Survival

**Figure 5:**
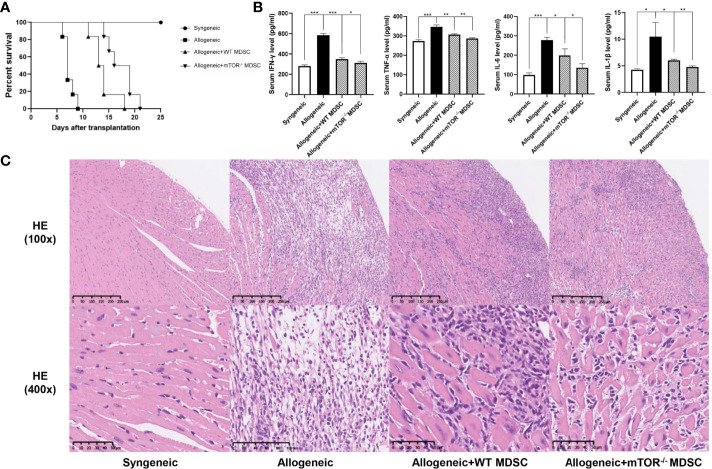
Infusion of mTOR^−/−^ M-MDSCs protected cardiac transplantation allografts. **(A)** Cardiac transplantation allograft survival rates. **(B)** ELISA analysis of IFN-γ, TNF-α, IL-6, and IL-1β in different groups. **(C)** H&E heart staining (n = 5, ^*^P < 0.05, ^**^P < 0.01, and ^***^P < 0.001).

To verify the RNAseq results, BALB/c to B6 cervical cardiac transplantation mouse models were established with the infusion of WT or mTOR^-/-^ M-MDSCs into the recipient mice. mTOR^−/−^ M-MDSC treatment significantly extended cardiac allograft survival compared to WT M-MDSCs (**Figure 5A**). ELISA was then employed to examine the inflammatory cytokines in the recipient serum samples and found that IFN-γ, TNF-α, IL-6, and IL-1β levels were all markedly downregulated in allogeneic HTx mice treated with mTOR^−/−^ M-MDSCs compared to the WT M-MDSC treatment (**Figure 5B**). In addition, H&E staining showed that mTOR^-/-^ M-MDSCs improved inflammatory cell infiltration and tissue damage in the hearts of allogeneic HTx mice (**Figure 5C**). These results indicated that mTOR^-/-^ M-MDSCs had a better potency in extending allograft survival, reducing inflammatory cytokines, and protecting tissues.

### The mTOR^−/−^ M-MDSC-Induced Immune Tolerance in HTx Mice

**Figure 6:**
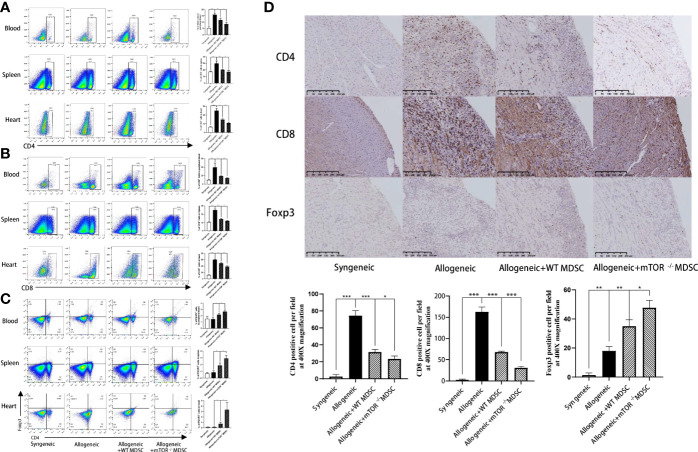
Infusion of mTOR^−/−^ M-MDSCs reduced CD4^+^ and CD8^+^ T cells and increased Tregs in blood, spleen, and heart. **(A)** Proportion of CD4^+^, CD8^+^ T cells, and Foxp3^+^ Tregs in blood. **(B)** Proportion of CD4^+^, CD8^+^ T cells, and Foxp3^+^ Tregs in spleen. **(C)** Proportion of CD4^+^, CD8^+^ T cells, and Foxp3^+^ Tregs in heart. **(D)** CD4, CD8, and Foxp3 immunohistochemistry staining in cardiac allograft with semi-quantification analysis (400×; n = 5, ^*^P < 0.05, ^**^P < 0.01, and ^***^P < 0.001).

Recipient mouse T cell status was then measured to investigate the protective function of mTOR^−/−^ M-MDSCs. Flow cytometry analysis demonstrated that the proportions of CD4^+^ and CD8^+^ T were decreased, while Treg populations were greatly increased in the peripheral blood, spleens, and hearts of mTOR^−/−^ M-MDSC-treated HTx mice (**Figures 6A–C**). Similar results were observed by immunohistochemistry analysis (**Figure 6D**). Together, these results suggested that infusion of mTOR^−/−^ M-MDSCs ameliorated T cell response and favor Tregs.

## Discussion

The mTOR is an evolutionary conserved serine-threonine kinase that senses various environmental stimuli in most cells primarily to control cell growth, cellular proliferation, and immunosuppressive function (19). In TME, mTOR activity in a subset of cells within the tumor mass can mediate MDSC accumulation (20). Welte et al. have found that oncogenic mTOR signaling recruits MDSCs to promote tumor initiation (21). Our previous study indicated that using rapamycin to inhibit the mTOR signaling pathway in MDSCs increases cell immunosuppressive function in AKI mouse models (13). However, whether mTOR regulates MDSCs in organ transplantations remains unclear. Thus, cervical cardiac transplantation models were used to investigate the function of mTOR-deficient M-MDSCs and to analyze mTOR regulation in MDSC proliferation and differentiation.

The present study used lyz-Cre-mTOR-flox mice to reveal the function of mTOR deficiency in myeloid cells, in which cells from other sources could still synthesize mTOR. The mTOR deficiency increased the proportion of endogenous M-MDSCs, while decreasing G-MDSCs in the spleens and hearts of Lyz-mTOR mice. Because G-MDSCs are considered to be immature MDSCs (1), it seems that mTOR deletion may have a role in promoting G-MDSC differentiation to M-MDSCs. Research also indicates that mTOR deficiency may result in more recruitment of myeloid-derived cells into organs. Results were similar to those in the present study in rapamycin-treated mice, as the total MDSC population in spleens was increased (12). In addition, spleen and heart M-MDSCs were isolated from WT and Lyz-mTOR mice and co-cultured with CFSE-labeled T cells to evaluate the immunosuppressive function of endogenous mTOR^−/−^ M-MDSCs. Both M-MDSC sources had a stronger suppressive function in inhibiting T cell proliferation, indicating that mTOR deficiency enhanced the immunosuppressive function of M-MDSCs. Our results are consistent with another study, which demonstrated the increased number of M-MDSCs from spleens of rapamycin-treated HTx mice and enhanced immunosuppressive ability to inhibit CD4^+^ T cell proliferation (12). However, Wu et al. have reported a contradictory conclusion that inhibition of mTOR by rapamycin inhibited the number and function of M-MDSCs in the spleen of alloskin-grafted mice, although the proportion of M-MDSCs in alloskin was not investigated (16). The discrepancy in mTOR inhibition in M-MDSCs may depend on microenvironment specificity in different disease models. Sun et al. have demonstrated that M-MDSCs with a high expression of CCR9 became the predominant type after MDSCs accumulated in the endometrium, resulting in the inhibition of autologous T cell activity in an immunoinflammatory microenvironment (22). This study suggested that immune cell migration from lymphoid organs to target tissues may largely rely on the expression of surface chemokine receptors, which can be recruited by their chemokine ligand secreted in the microenvironment. Nevertheless, further study on MDSC function is still needed.

Inhibition of the amino acid-responsive mTOR kinase complex is a key signal for autophagosome biogenesis, primarily through activation of the ULK kinase complex that occurs in conjunction with that of the PIK3C3-BECN1-Atg14 complex (23). Tumor-infiltrating autophagy-deficient M-MDSCs demonstrated impaired suppressive activity *in vitro* and *in vivo*, whereas M-MDSCs exhibited impaired lysosomal degradation, thereby enhancing surface expression of MHC class II molecules and resulting in efficient activation of tumor-specific CD4^+^ T cells (17). In addition, degradation of cytoplasmic components through starvation-induced autophagy regenerates amino acids that are used in the tricarboxylic acid cycle to produce energy that the cells need to survive, thus allowing them to avoid death  (24). The present study found that the intracellular autophagy levels were up-regulated in mTOR-deficient M-MDSCs, which explained the enhanced cell differentiation and immunosuppressive function.

A previous study has demonstrated that using rapamycin in mouse cardiac transplantation models enhanced the proliferation of MDSCs, which prolonged allograft survival (12). However, the rapamycin action mechanism remains unclear. Thus, RNAseq was used to investigate the potential function of mTOR-deficient M-MDSCs. KEGG pathway analysis showed that induced M-MDSCs from Lyz-mTOR mice might have a role in preventing allograft rejection. Based on these analysis results, Lyz-mTOR mouse BM was used to induce mTOR^−/−^ M-MDSCs, which were then infused into cardiac transplantation recipient mice. The results showed that mTOR^−/−^ M-MDSCs did not only prolong allograft survival, but also reduced T cell infiltration and increased Treg populations in the allografts. However, the molecular mechanism involved in the prone differentiation of M-MDSCs with mTOR deficiency genetically remains unclear. Recently, Yo et al. have shown that a combined inhibition of glutamine metabolism and mTOR significantly suppressed CD4^+^ T cell activation-mediated arthritis in mice better than each monotherapy, implying the essential role of cellular metabolism in immune cell mTOR signaling (25). Another study reported that inhibition of mTOR activity by SIRT1- and HIF-1α-associated metabolism participated in regulating the differentiation and function of Th9 cells (26). Both studies focusing on cellular metabolism are concordant with our data analyzed by RNAseq to some extent, showing that retinol, ascorbate, and aldarate metabolism might be involved in mTOR-mediated MDSC differentiation. The exact mechanism requires further verification and study.

In conclusion, the present study found that mTOR deficiency enhanced the immunosuppressive function of M-MDSCs both *in vitro* and *in vivo*, which might be correlated with the increased levels of intracellular autophagy. In addition, mTOR-deficient M-MDSCs prolonged cardiac allograft survival and induced immune tolerance by increasing Treg population. These findings explained the function of mTOR in MDSCs, demonstrated that mTOR^−/−^ M-MDSCs had a stronger immunosuppressive ability, and suggested that infusing with mTOR^−/−^ M-MDSCs reduced allograft rejection and induce immune tolerance. These results might be of benefit for clinical translation of cell therapy in organ transplantation.

## Data Availability Statement

The datasets presented in this study can be found in online repositories. The name of the repository and accession number can be found below: National Center for Biotechnology Information (NCBI) Gene Expression Omnibus (GEO), https://www.ncbi.nlm.nih.gov/geo/, GSE167594.

## Ethics Statement

The animal study was reviewed and approved by Animal Ethical Committee of Zhongshan Hospital, Fudan University.

## Author Contributions

YZ and RR conceived the project, designed the project and approved the final manuscript. JWL drafted the manuscript. JWL, JC, MZ, CZ, YQ and JJL conducted the experiments. RW and TY analyzed RNAseq data. All authors contributed to the article and approved the submitted version.

## Funding

This work was supported by the National Key R&D Program of China (2018YFA0107501 to RR), National Natural Science Foundation of China (81400688 to YZ, 81770747 and 81970646 to RR).

## Conflict of Interest

The authors declare that the research was conducted in the absence of any commercial or financial relationships that could be construed as a potential conflict of interest.

## Supplementary Material

Supplementary Figure 1 The expression of mTOR in M-MDSCs induced from BM of WT and Lyz-mTOR mice in mRNA level.
