# Supplementary figures and images for: The mTOR Deficiency in Monocytic Myeloid-Derived Suppressor Cells Protects Mouse Cardiac Allografts by Inducing Allograft Tolerance
Source: Front Immunol. 2021 Apr 9;12:661338. doi: 10.3389/fimmu.2021.661338 (PMC8062712; doi:10.3389/fimmu.2021.661338)

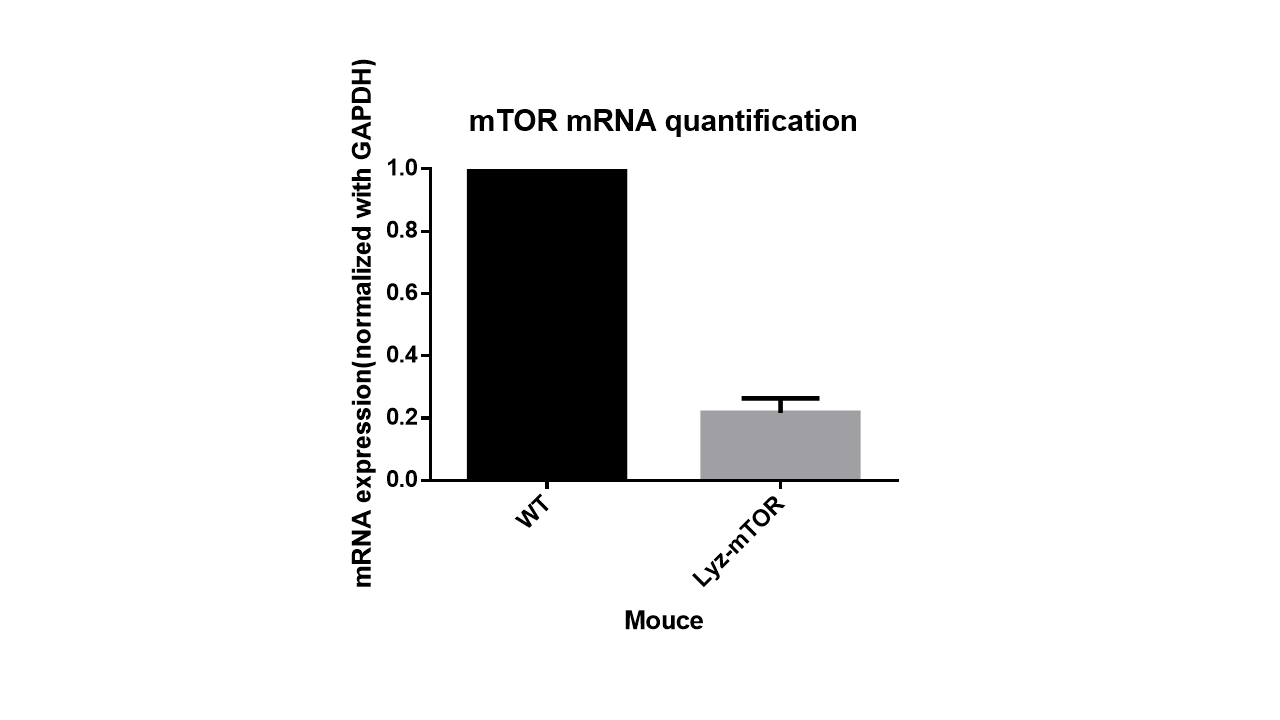

Supplement: Supplementary Figure 1 — The expression of mTOR in M-MDSCs induced from BM of WT and Lyz-mTOR mice in mRNA level. [file Image_1.tif]
